# Interaction of Micro- and Nanoplastics with Enzymes: The Case of Carbonic Anhydrase

**DOI:** 10.3390/ijms25179716

**Published:** 2024-09-08

**Authors:** Gregorio Polo, Francesca Lionetto, Maria Elena Giordano, Maria Giulia Lionetto

**Affiliations:** 1 Department of Mathematics and Physics, University of Salento, Via per Arnesano, 73100 Lecce, Italy; gregorio.polo@unisalento.it; 2 Department of Engineering for Innovation, University of Salento, Via per Monteroni, 73100 Lecce, Italy; francesca.lionetto@unisalento.it; 3 Department of Environmental and Biological Sciences and Technologies (DiSTeBA), University of Salento, Via per Monteroni, 73100 Lecce, Italy; elena.giordano@unisalento.it; 4 National Biodiversity Future Center (NBFC), 90133 Palermo, Italy

**Keywords:** carbonic anhydrase, microplastics, nanoplastics, enzymes, toxicological effects, ecotoxicological effects, biomarker, plastic degradation, esterase activity, emergent pollutant

## Abstract

Microplastics (MPs) and nanoplastics (NPs) have emerged as significant environmental pollutants with potential detrimental effects on ecosystems and human health. Several studies indicate their interaction with enzymes; this topic represents a multifaceted research field encompassing several areas of interest from the toxicological and ecotoxicological impact of MPs and NPs on humans and wildlife to the biodegradation of plastics by microbial enzymes. This review aims to provide a critical analysis of the state-of-the-art knowledge of the interaction of MPs and NPs on the enzyme carbonic anhydrase (CA), providing recent insights, analyzing the knowledge gaps in the field, and drawing future perspectives of the research and its application. CA is a widespread and crucial enzyme in various organisms; it is critical for various physiological processes in animals, plants, and bacteria. It catalyzes the reversible hydration of CO_2_, which is essential for respiration, acid–base balance, pH homeostasis, ion transport, calcification, and photosynthesis. Studies demonstrate that MPs and NPs can inhibit CA activity with mechanisms including adsorption to the enzyme surface and subsequent conformational changes. In vitro and in silico studies highlight the role of electrostatic and hydrophobic interactions in these processes. In vivo studies present mixed results, which are influenced by factors like particle type, size, concentration, and organism type. Moreover, the potentiality of the esterase activity of CA for plastic degradation is discussed. The complexity of the interaction between CA and MPs/NPs underscores the need for further research to fully understand the ecological and health impacts of MPs and NPs on CA activity and expression and glimpses of the potentiality and perspectives in this field.

## 1. Introduction

**Figure 1:**
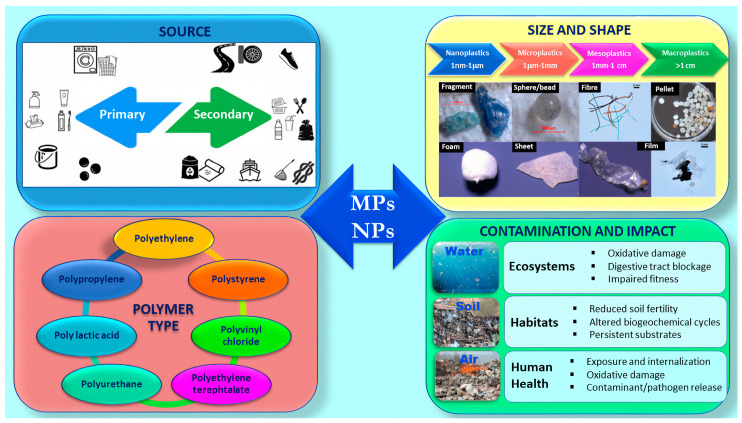
Source, size, shape, polymer type, contamination and impact of microplastics and nanoplastics. Reproduced from [6,7,8] with permission from Elsevier (Copyright 2022).

Microplastics (MPs) and nanoplastics (NPs) have recently gained attention as emerging contaminants in the sea, freshwater, sediments, soils, and the atmosphere [1] with potentially hazardous impacts on ecosystems and human health. MPs are polymer particles ranging from 1 µm to 5 mm, while NPs have sizes lower than 1 µm. MPs/NPs represent 92% of the 5.25 trillion plastic particles on the ocean surface even if the absolute volumes of plastic debris across different marine environments remain largely underestimated or unknown [2]. According to their source, MPs/NPs can be classified as primary or secondary [3], as sketched in Figure 1. Primary MPs/NPs are manufactured directly by industries as raw materials for other products such as microbeads for personal care products (exfoliants, toothpaste, and cosmetics), pellets for plastic manufacturing processes, powders for abrasive cleaning agents and other industrial applications, fibers released from synthetic textiles during washing, etc. [3]. Secondary MPs/NPs result from the breakdown of larger plastic debris due to weathering processes as a consequence of UV radiation, mechanical abrasion, and chemical degradation [4,5]. As reported in Figure 1, a wide range of MP/NP shapes (including microbeads, fragments, fibers, foams, and films) and compositions have been detected in different environments, such as aquatic environments, soil, and air.

The small size of MPs and NPs and their ability to adsorb other contaminants make them potentially harmful to exposed organisms. The widespread distribution of MPs and NPs in the environment raises concerns about their long-term effects on ecosystems and human health (Figure 1).

Particularly, NPs raise great concern due to their potential to interact with biological macromolecules such as proteins and nucleic acids thanks to their nanometric dimensions. The interaction of MPs and NPs with enzymes has gained significant interest in this field due to the growing environmental and health concerns associated with plastic pollution. It represents a multifaceted research field encompassing several areas of interest from the toxicological and ecotoxicological impact of MPs and NPs on humans and wildlife with implications for human and environmental health to the biodegradation of plastics by microbial enzymes with implications for the development of new biotechnologies and a significant impact on environmental sustainability. Concerning this last aspect, several types of enzymes, such as hydrolases (esterases, cutinases, and lipases) and oxidoreductases, have been discovered in some microbic and insect species to help plastic biodegradation [9,10,11,12,13,14,15]. This paved the way for the development of many enzyme-based techniques for environmentally friendly plastic degradation [16,17].

Considerable international interest is also growing in the research field of the toxicology and ecotoxicology of the interaction of MPs and NPs with enzymes and the consequent alteration of enzyme activity and expression with implications for human and wildlife health. Oxidative stress induction, metabolic disorder, and neurotoxicity are among the toxicological effects caused by MPs and NPs exposure in which the alteration of enzyme activity and expression was found to be involved [18,19,20]. For instance, exposure to NPs has been proven to alter the activity of antioxidant enzymes in aquatic organisms by promoting the formation of ROS and subsequent oxidative damage [20,21]. Moreover, exposure to MPs and NPs was found to decrease digestive enzyme activity (lipase, trypsin, and lysozyme) with consequent harmful impact on the intestinal functionality of invertebrates and fish [18,22,23,24]. The activity of lipase was found to decrease in the presence of polystyrene NPs, contributing to altered digestive function and growth in juvenile groupers [25]. Exposure to micro- and nanoplastics induced the inhibition of acetylcholinesterase activity and altered neurotransmitter levels with consequent behavioral changes in aquatic organisms [19,26]. Research in this area is ongoing to understand these interactions’ full extent and implications.

Carbonic anhydrase (CA) is one of the most widespread enzymes in nature, catalyzing the reversible conversion of CO_2_ to HCO_3_^−^. It plays a key role in the physiology of animals, plants, and bacteria. In animals, CA isoforms are fundamental for respiratory gas exchange, acid–base regulation, metabolism, osmoregulation, calcification, biomineralization and bone resorption, signal transduction, and cellular defenses against oxidative stress thanks to the pivotal role played by bicarbonate and protons in organism physiology [27]. In plants, algae, and some bacteria, CAs are crucial for photosynthesis [27,28]. In recent years, several studies have documented the sensitivity of the activity and expression of CA to MPs and NPs in several organisms through in silico, in vitro, and in vivo approaches. Given the enormous importance of this enzyme in the physiology of living organisms and its widespread occurrence in nature, the study of the effects of widely distributed pollutants like MPs and NPs on this enzyme is of great relevance for implications on human health and the environment. It could provide valuable insights into the environmental and health impacts of plastic pollution; it may reveal new mechanisms of toxicity and provide effect-based tools for assessing the ecotoxicity of MPs and NPs in the environment. Another aspect of CA that deserves particular interest is the exhibition of some esterase activity by CA other than its well-known catalytic role in the hydration of carbon dioxide. In general, esterases from various microorganisms proved to be able to degrade plastics by breaking down ester bonds into monomers and oligomers [29,30]. Although the literature on the involvement of CA esterase activity in plastic degradation is lacking, in perspective, this represents an intriguing research field for the potential biotechnological and environmental implications.

Considering all these issues, CA represents an enzyme with multiple facets raising multiple relevant interests for the study of interactions with MPs and NPs. This review aims to provide a critical overview of the state-of-the-art knowledge of the interaction of MPs and NPs with CAs. It provides recent insights, analyzes the knowledge gaps in the field, and draws future perspectives on the research and its application.

## 2. Interaction of MPs and NPs with Enzymes

The interaction between enzymes and micro/nanoplastics is a rapidly growing area of research with a range of interdisciplinary focuses such as the enzymatic degradation of plastics and the toxicological and ecotoxicological impact on humans and wildlife.

### 2.1. Enzymatic Degradation of MPs and NPs

The enzymatic degradation of synthetic polymers is an appealing and promising approach to reducing environmental pollution from plastic materials [16]. Several types of enzymes converting polymers into monomers have been discovered to help the biodegradation of plastic materials [11,12,13]. Microbial enzymes can easily hydrolyze PET (polyethylene terephthalate) and PUR (polyurethane), which are provided with ester bonds in their backbones. Therefore, these plastic materials are more susceptible to biodegradation than other polymers, like PE (polyethylene), and PS (polystyrene), which have carbon chains in their backbone [31,32,33]. Enzymes involved in plastic biodegradation can be primarily distinguished into two groups, such as extracellular and intracellular enzymes [11,32,34,35]. Extracellular enzymes depolymerize the long-chain MP polymers into a mixture of small-chain oligomers, dimers, and monomers [12,13,33]. These enzymes show a wide range of activities, including oxidative and hydrolytic activity [36]. On the other hand, intracellular enzymes are involved in the assimilation and mineralization of the oligomers and monomers through aerobic and anaerobic processes whose end products are represented by CO_2_ and H_2_O [11,31]. The main classes of enzymes involved in the biodegradation of MPs and NPs are hydrolases and oxidoreductases. The class of hydrolases includes lipases, cutinases, and proteases. Lipases are mostly extracellular enzymes catalyzing the hydrolysis of insoluble triacylglycerides to free fatty acids and glycerol [37]. Particularly, microbial lipase has attracted greater industrial interest than those derived from plants and animals thanks to its functional stability at extreme pH and temperature conditions [38]. Bacterial lipases can hydrolyze aliphatic polyesters as well as aliphatic-aromatic co-polyesters [39]. Cutinases are extracellular enzymes able to hydrolyze numerous polyester polymers [32,40]. Bacterial and fungi cutinases [41,42] find major applications in plastics degradation. Esterases represent a various group of hydrolases catalyzing the cleavage and formation of ester bonds and are widely distributed in animals, plants, and microorganisms [43,44]. They are able to degrade biodegradable plastic materials by breaking down ester bonds into monomers and oligomers [29,30]. Proteases catalyze the hydrolysis of the peptide bond present in the polypeptide chain of amino acids [42,45]. Proteases from species such as *Pseudomonas chlororaphis* and *Pseudomonas fluorescens* have been reported to degrade polyester PUR [46].

Although plastic-degradation enzymes have considerable relevance for plastic degradation and reducing environmental pollution from plastic material and are attracting great attention for their application, little information is available to date on their biochemical properties and their structural characteristics [9]. The study of enzymes involved in plastics degradation is an active and promising field of research, but further work is still required for the development of this technology and its wide-scale application.

### 2.2. Toxicological and Ecotoxicological Effects of MPs and NPs on Enzymes

**Figure 2:**
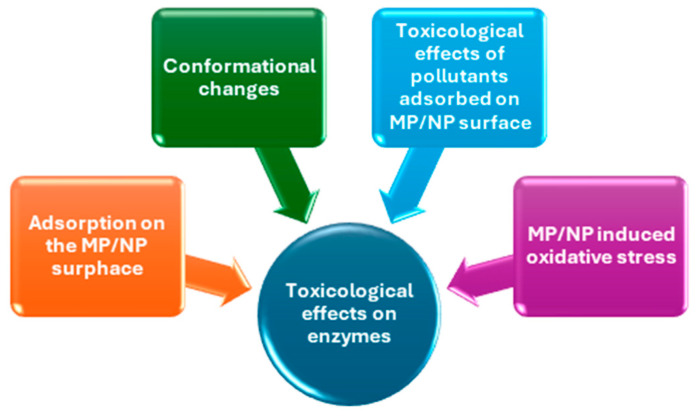
Mechanisms of toxicological interaction between MPs/NPs and enzymes.

As the awareness of MPs and NPs in the environment increases, so does the interest in understanding their impact on humans and wildlife. Several studies demonstrated that MPs and NPs can interact with enzymes affecting their activity and expression. Particularly, NPs are more likely to interact at the molecular level due to their small size and high surface area. The mechanisms of interaction can be different according to the specific enzymes and particles. In general, they include the adsorption of the enzyme on the MP/NP surface, potentially altering the enzyme’s active site and reducing its catalytic activity (Figure 2). Possible conformational changes in enzyme structure can occur, affecting the enzymatic functionality. As recently demonstrated by Hollóczki et al. [47], different types of NPs (polyethylene, polypropylene, polyethylene terephthalate, and nylon-6,6) chosen in the dimensional range of 5 nm, which is expectedly the lower end of the size range that is available in the environment, can change the functionally secondary structure of proteins and denature them, interfering with the folding of the protein.

Moreover, the interaction of NPs with enzymes is made more complex by the leaching of monomers, additives, or other pollutants adsorbed to the nanoplastics themselves (Figure 2). Researchers have widely demonstrated in the literature the sorption of organic and inorganic pollutants on MP/NPs dominated by different mechanisms, including hydrophobic interactions, π–π interactions, electrostatic interactions hydrogen bonding, and Van der Waals forces [48,49,50,51]. All these substances can potentially interact with enzymes, altering enzymatic activity.

In most cases, the interaction between MPs/NPs and enzymes causes an inhibition of enzymatic activity. Nanoplastic-induced enzyme inhibition has been documented for several enzymes, including acetylcholinesterase, lipase, alkaline phosphatase, esterase, protease, and catalase in various organisms [52] with consequent disruptions of metabolic processes and overall organism health. For instance, polystyrene NPs (50 nm) have been shown to inhibit acetylcholinesterase activity in *Danio rerio* larvae, and this effect was associated with reduced locomotion activity [53]. Exposure to polystyrene NPs (50 nm) has been shown to reduce lipase activity by changing the secondary structure and disturbing the essential open conformation following the adsorption of the enzyme on the particles [54]. Polystyrene NPs with different surface functionalization (plain (PS), amine (PS-NH_2_), and carboxy (PS-COOH)) and different sizes (100 and 500 nm) were able to inhibit the esterase-like activity of human serum albumin, which plays a role in metabolizing drug/toxic compounds [55].

An area of great interest in this field is the impact of MPs/NPs on antioxidant enzymes, which play a crucial role in mitigating oxidative stress [56]. Oxidative stress is, in turn, a fundamental underlying mechanism for further toxic effects induced by MPs/NPs in a majority of organisms [57]. Exposure to NPs leads to an increased production of reactive oxygen species (ROS), which in turn affects the activity of antioxidant enzymes such as superoxide dismutase (SOD), catalase (CAT), and glutathione peroxidase (GPx) [58,59,60,61,62]. High concentrations of MPs/NPs generally decrease the activities of antioxidant enzymes, while low concentrations may initially increase these activities before leading to suppression at higher doses [58,59,63]. Smaller nanoplastics (e.g., 100 nm) and those with specific surface modifications (e.g., amide-modified) exhibit higher toxicity and more pronounced effects on antioxidant enzyme activities compared to larger particles [59,62,64,65]. The toxicity and oxidative stress induced by nanoplastics are size-dependent with smaller particles causing more significant adverse effects [62,64]. Nanoplastic exposure activates stress-related pathways, including the MAPK-HIF-1/NFkB pathway, which influences the expression of genes related to antioxidant defense [58,61,62]. The expression of genes encoding antioxidant enzymes (e.g., SOD, CAT, GPx) is generally upregulated in response to MP/NP-induced oxidative stress, though this response can vary with concentration and exposure duration [21,58,61]. Therefore, MPs/NPs can significantly affect the activity of antioxidant enzymes across various organisms. The concentration, size, and surface properties of the nanoplastics influence these effects. Generally, exposure to nanoplastics leads to increased oxidative stress, which initially may upregulate antioxidant enzyme activities but ultimately suppresses them at higher concentrations. The specific responses can vary among different species and depend on the particular characteristics of the NPs involved.

The toxicological and ecotoxicological interaction of micro- and nanoplastics with enzymes is a critical area of research with significant implications for human health and for environmental monitoring and management; however, much research in this field still needs to be conducted. While there is growing interest in the effects of MPs and NPs on enzymes, the specific mechanisms of effect in the various organisms, the following toxicological effects at the whole organism level, and the factors and conditions influencing these effects are not yet fully understood, and several gaps in the knowledge are present in this field.

Moreover, the research field of the toxicological and ecotoxicological effects of MPs/NPs on enzymes also interests other research fields such as the research on the enzymes degrading plastics. Indeed, in light of the current knowledge of the toxicological and ecotoxicological effects of MPs/NPs on enzymes in various organisms, an important ecotoxicological aspect that deserves to be carefully explored in the future is the eventual toxicological effects of MPs/NPs on microbial enzymes involved in plastic degradation. The eventual toxicological interaction between these enzymes and MPS/NPs, and whether MPs/NPs inhibit microbial degrading enzymatic activities, is a critical area of investigation.

## 3. Carbonic Anhydrase

Among enzymes, CA is one of the most widespread in nature. It is a ubiquitous metalloenzyme present in prokaryotes and eukaryotes; it catalyzes the bidirectional conversion of carbon dioxide (CO_2_) and water (H_2_O) into bicarbonate (HCO_3_^−^) and protons (H^+^). CA isoforms are classified into eight genetically distinct and unrelated superfamilies: α-CA expressed in animals, protozoa, plants, algae, bacteria, fungi, β-CA expressed in plants, algae, archaea, bacteria, fungi, γ-CA expressed in archaea, bacteria, plants, δ-CA, ζ-CA and θ-CA expressed in marine diatoms, η-CA expressed in protozoa, and ι-CA expressed in diatoms and bacteria [28,66,67]. Due to the pivotal role played by HCO_3_^−^ and H^+^ in biological processes, CA catalytic activity is involved in several physiological processes.

In all of the CA superfamilies, the metal ion is fundamental for catalysis, as the apoenzyme is devoid of activity. The metal is Zn^2+^ for all classes, but other transition metals have been demonstrated to bind to the catalytic site as physiologically relevant metal cofactors or displacers of the native cofactor [27]. All CA isoforms catalyze the reversible hydration of CO_2_ to HCO_3_^−^ and H^+^ through a metal-hydroxide [Lig3M^2+^(OH)^−^] mechanism [68], which involves the reaction between CO_2_ and the OH^−^ bound to the zinc ion and yields a coordinated HCO_3_^−^ ion, which in turn is displaced from the metal by H_2_O. Lig3 is represented by three key amino acid residues: three histidines in α, γ, and δ CAs, one histidine and two cysteines in β and ζ Cas, and two histidines and one glycine in η CA [27]. A fourth amino acid (represented by His 64 in human CAII) not directly included in the active site helps the catalysis by acting as a “proton shuttle” which allows the H^+^ transfer from the water molecule bound to the metal in the active site to buffer molecules outside the active site. This step guarantees the reaction of the metal-bound OH^−^ with CO_2_ to produce HCO_3_^−^.

CA activity is known to be sensitive to several chemical agents. The research field of CA activity inhibition encompasses several research fields from human health and clinics, thanks to the development of several agents targeting specific CA isoforms for specific therapeutic applications [68,69], to environmental monitoring thanks to the discovery of the sensitivity of CAs to several environmental pollutants that are both organic and inorganic [27,70,71,72,73,74,75,76,77,78]. Cadmium (Cd), lead (Pb), mercury (Hg), and other heavy metals can bind to the active site of carbonic anhydrase or interact with its structure, inhibiting its enzymatic activity [27]. These metals can also induce oxidative stress, leading to changes in CA expression as part of the cellular stress response [76,77]. Certain pesticides and herbicides can affect carbonic anhydrase activity [75]. These chemicals may bind to the enzyme or interfere with its expression. Some studies have shown that organophosphates and carbamates pesticides can inhibit CA activity in various aquatic organisms, impacting their respiration and osmoregulation [75,79]. Also, polyaromatic hydrocarbons (PAHs), pharmaceuticals, and other organic pollutants can alter CA expression and activity [76,80]. In the research field of the sensitivity of CA to environmental pollutants, the study of the effect of MPs and NPs on carbonic anhydrase is an emergent research topic. Recent scientific literature has started to explore the interactions between MPs and NPs and carbonic anhydrase [81], suggesting an emergent and rapidly evolving research area.

### 3.1. Sensitivity of CA Activity and Expression to Micro- and Nanoplastic Exposure

In the present review, a comprehensive literature analysis of the sensitivity of CA activity and expression to MPs and NPs exposure was carried out from various sources such as ScienceDirect, Scopus, and Google Scholar in the last 10 years from 2014 to 2024 using the following keywords: “Microplastic” or “Nanoplastic” and “Carbonic Anhydrase”.

The results of the search are reported in Table 1 and Table 2 and were organized based on the type of organism, species, size, and type of MPs or NPs, concentration and time of exposure used, other contaminants present with relative concentration, type of tissue, type of analysis, and toxicological effect on CA. Plastic particles sized between 1 μm and 5 mm were classified as MPs, while particles smaller than 1 μm were classified as NPs.

**Figure 3:**
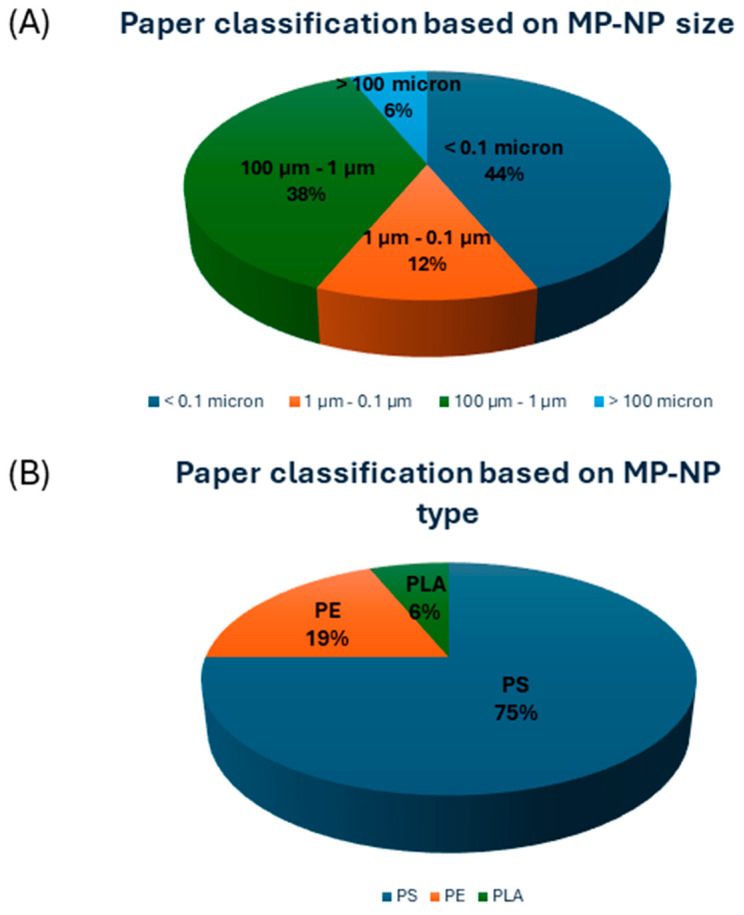
Paper classification based on MP-NP size (**A**) and type (**B**).

**Figure 4:**
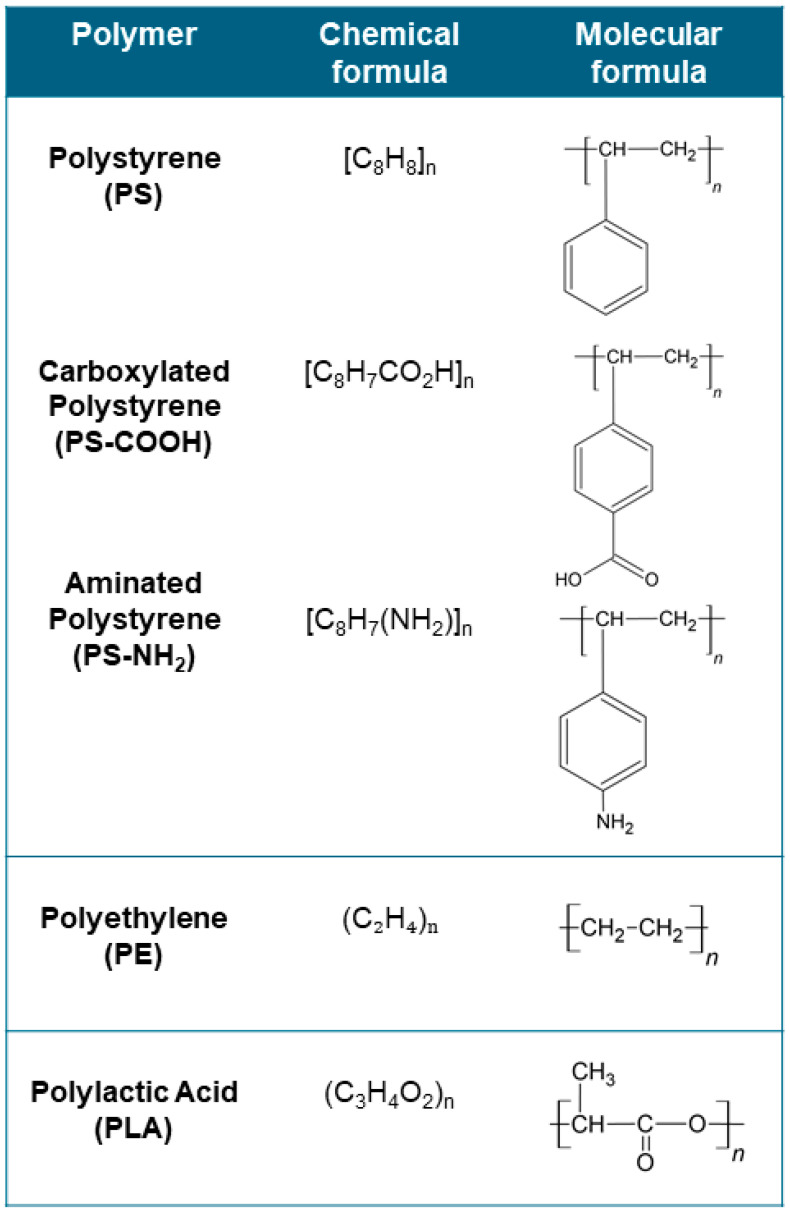
Chemical and molecular formula of the polymers used for studying the interaction with CA enzyme.

The research identified 16 papers which included six papers on mollusks (particularly five on bivalves and one on gastropods), four on humans, two on teleosts, one on arthropods, one on dinoflagellate, one on diatoms, and one on cyanobacteria. Among these papers, 44% were related to NPs and 56% were related to MPs microplastics according to the particle classification as reported in Figure 3A. Additionally, most of the works available considered the effects of polystyrene MPs and NPs (75%), three investigated polyethylene (19%), and only one study investigated polylactic acid (PLA) microplastics (see Figure 3B). The chemical and molecular structure of the plastic materials studied to date for the interaction with CA enzymes is reported in Figure 4. Polystyrene and polyethylene polymers are among the most widely used plastics. They are synthesized for various applications, including food packaging, personal care products, and building insulation. Both polystyrene and polyethylene are highly frequently detected forms of MPs and NPs in aquatic and terrestrial environments [97]. PLA is the most popular bio-based polymer, being a biodegradable hydrolysable aliphatic semicrystalline polyester produced through the direct condensation reaction of its monomer, lactic acid, as the oligomer, and followed by ring-opening polymerization of the cyclic lactide dimer [98].

The literature analyzed in this work encompasses different experimental approaches including in silico, in vitro, and in vivo studies.

#### 3.1.1. In Vitro and In Silico Studies

Although limited, in vitro and in silico studies allow an understanding of the molecular interaction mechanisms between CA and MPs and NPs (Table 1). In particular, Assarsson et al. [82] found that polystyrene NPs (with a hydrodynamic diameter between 30 and 60 nm) inhibited the esterase activity of human carbonic anhydrase II (HCAII) in vitro. They demonstrated that the interaction between NPs and CA occurs through an adsorption process of the enzyme on the nanoparticles followed by protein conformational changes. The adsorption process was influenced by nanoparticle type, size, and functionalization. Negatively charged plastic particles such as carboxyl-modified polystyrene nanoparticles (PSCOOH^−^) strongly and quickly inhibited the activity of CA (with an IC_50_ value in the nanomolar range) compared to positively charged particles such as PSNH_2_ in which no CA adsorption was observed.

The analysis of the interaction between CA and PSCOOH^−^ was deepened in the work of Assarsson et al. [83], in which the activity and adsorption of three variants of human carbonic anhydrase with similar topology but variations in charge and stability (HCAI, HCAII, trHCAII, a truncated version of HCAII lacking the 17 N-terminal amino acid residues) were studied in the presence of PSCOOH^−^—of different sizes ranging from 25 to 114 nm. The study found that the adsorption of these proteins onto nanoparticles leads to a loss of enzymatic activity. Non-electrostatic contributions included hydrophobic interactions with the particle surface representing the driving forces for protein adsorption in the case of HCAII. On the other hand, electrostatic forces represented the driving force for the interaction between human carbonic anhydrase I and N-terminal truncated human carbonic anhydrase II and PS nanoparticles. This suggests that the physicochemical properties of the proteins heavily influence the adsorption of CA proteins onto nanoparticles. Particle size affected protein adsorption by changing the nanoparticle–protein contact area but also by modulating the lateral interactions among the adsorbed proteins, since lateral repulsive interactions between adsorbed proteins are geometrically minimized due to the increase in particle curvature with the decrease in particle size. All three HCA variants were completely inhibited upon adsorption to PS carboxylated nanoparticles. The mechanism of inhibition was attributed to the transition of the CA protein toward a molten globule-like state lacking catalytic activity. It is known that CA has a folding intermediate between the native and the unfolded state known as the molten-globule state [99,100]. This intermediate state shows a compact core retaining most of the secondary structure of the native state, while the tertiary structure is partially distorted and lacks enzymatic activity. Therefore, the authors attributed the loss of enzymatic activity to a transition toward a molten globular-like state upon the adsorption on PSCOOH^−^ nanoparticles as observed already by Nasir et al. [81]. The authors also suggest an alternative explanation to the activity inhibition: the access of the substrate to the active site might be partially hindered due to the orientation of the protein on the particle surface.

Moreover, a contribution to the understanding of the nature of the molecular interaction between CA protein and MP/NPs comes from an in silico study carried out on CA in the diatom *Chaetoceros neogracile* through docking analysis [1]. This CA belongs to the δ-CA family, and it is involved in the carbon-concentrating mechanisms that diatoms use to efficiently acquire and utilize inorganic carbon for photosynthesis. The docking analysis revealed a strong affinity of CA toward the cyclic aromatic rings of polystyrene, suggesting the potential mechanism by which PSMPs can impact the photosynthesis process of microalgae.

Overall, the in vitro and in silico studies to date available, although limited and carried out only on polystyrene NPs, outline the great potential of NPs to directly interact with CAs molecules and inhibit the catalytic activity indicating electrostatic and/or hydrophobic interactions as drivers of the adsorption process according to the NP chemical nature, surface functionalization and CA isoform. The acquisition of the molten globule-like state of the adsorbed enzyme seems to be a reasonable explanation for the catalytic activity inhibition. However, future studies are required to clarify better the mechanism of action of different NPs on the various CA isoforms present in nature.

#### 3.1.2. In Vivo Studies

In vitro studies are useful for understanding the interaction mechanisms of MPs and NPs with CA and for an early toxicological assessment. However, they do not provide information about absorption, distribution, metabolism, excretion, and effects on target sites in the body. Therefore, for a more realistic evaluation of the sensitivity of CA activity and expression to MPs and NPs exposure, in vivo studies were considered. In vivo studies allow us to observe whole-organism responses, more realistic exposure conditions, and the integrated response of the interaction of MPs and NPs with multiple physiological systems. The currently available in vivo studies on the effects of MPs and NPs on CAs are reported in Table 2. These studies encompass various species, including cyanobacteria, dinoflagellates, mollusks, crustaceans, fish, and humans. Mollusks are the most represented taxon studied thanks to their responses to toxic contaminants, short life cycle, wide distribution, bioaccumulation capability for a wide range of pollutants, various life stages with different sensitivity, and broad use for standardized tests in water and sediment quality assessment as bioindicators [101]. Most in vivo studies have been conducted on polystyrene (PS) nanoplastics, few studies are available on polyethylene (PE), and only one study is available on PLA.

The effects induced by PS MP/NP on CA include the alteration of CA gene expression with upregulation assessed in the bivalve mollusks *Mytilus coruscus* [85] and *Mytilus galloprovoncialis* embryos [87] after exposure to PS MPs under short-term exposure. In addition to other physiological functions, in bivalve mollusks, CAs play significant roles in the biomineralization process, which is crucial for shell formation and maintenance. The induction of CA4, a glycosylphosphatidyl-inositol-anchored membrane isozyme involved in the homeostasis of carbon dioxide and bicarbonate, was observed after 24 h exposure to carboxylated PS NP in human embryos and induced pluripotent stem cells [91]. On the other hand, aminated polystyrene nanoparticles induced the downregulation of CA gene expression in *Mytilus galloprovincialis* embryos after 48 h of exposure (0.150 mg/L), highlighting the importance of NPs surface functionalization in the induction of toxicological responses.

Under long-term exposure to PS MPs, the CA tissue content was reduced in the mantle of the bivalve mollusk *Mytilus coruscus* as assessed by ELISA [86] after four weeks of exposure. In the case of the freshwater shrimp *Macrobrachium nipponense*, the exposure to increasing concentrations of PS NP induced a non-monotonic dose–response of CA gene expression in gills where CA plays a key role in ion transport and gas exchange. A significant upregulation of the gene was observed at the lower concentration tested (5 and 10 mg/L), and there was a decline of the induction at higher exposure concentrations (20 and 40 mg/L), which was presumably due to the toxic effect of a high accumulation of particles with time on cell functionality. Moreover, in the common harmful algal blooms-causing dinoflagellate *Alexandrium tamarense*, a significant inhibition of extracellular CA activity was observed within 96 h of exposure to PS MPs and NPs, which was accompanied by inhibited population growth, photosynthetic efficiencies, and inorganic nutrient uptake [89]. In this organism, CA_ext_ is responsible for providing carbon sources for central carbon metabolism (CCM) and maintaining intracellular pH stability. The effect on CA_ext_ was time-dependent and was more marked at the higher concentration tested (50 mg/L).

As for the effects of PE particles on CA, only three studies are available on the action of PE MPs on enzymatic activity, while no studies are available on the in vivo effects of PE NPs on CAs activity or expression. No significant effect was observed in two works carried out on the neotropical teleosts *Astyanax lacustris* (microplastic size: 100 and 200 microns) and *Prochilodus lineatus* (microplastic size in the range of 10 to 90 microns), respectively, during short-term exposure experiments [94,95]. In the third study on PE microplastics (microplastic size in the range of 10 to 90 microns) conducted on the freshwater snail *Pomacea canaliculata*, the inhibition of CA activity in the digestive gland was observed after 24 h of exposure, and stimulation of the activity measured in the mantle was observed after 24 and 72 h of exposure.

In addition to PS and PE MPs/NPs, only one study is available in the literature on the in vivo effect of PLA on CA [96]. The authors found a reduction in the intracellular CA content in the cyanobacteria *Microcystis aeruginosa* population exposed to PLA-MPs under long-term exposure. They attributed this result to an indirect effect of PLA arising from the enhanced PLA degradation following the interaction of the MPs with the cyanobacteria and, in turn, enhanced inorganic carbon availability for the organism and subsequent CA reduction as a compensatory response.

It is known that MPs and NPs can adsorb and transport other pollutants, serving as vectors for aquatic contaminants such as metals and polycyclic aromatic hydrocarbons (PAHs) [102]. Very few studies on CA and MPs and NPs exposure analyzed this aspect. The study of Campos et al. [95] considered the effect of low-density PE MPs preloaded with PAH for 96 h on the fish *Astyanax lacustris* on CA activity in the gills. The authors did not find any significant effect on CA after short-term exposure, although other metabolic alterations were detectable in the exposed fish. The same result was obtained by Roda et al. [94] on the fish *Prochilodus lineatus* exposed for 24 and 96 h to PE MPs preloaded with copper. The study of Yu et al. 2022 [86] considered the effects on CA in *Mytilus coruscus* of the multiple exposure to PS and another emerging pollutant represented by carbamazepine, which belongs to the class of pharmaceuticals released into the environment. In this case, a synergistic inhibitory effect was observed on CA expression after 4 weeks of exposure. In the bivalve mollusk *Laternula elliptica* [90], the specific induction of CA9 was detected after the contemporary exposure to PSCOOH NPs 5 µg/L and TiO_2_ 5 µg/L, highlighting the presence of a synergistic effect also in this case.

Overall, the available data on the in vivo effect of MPs/NPs are limited in number, related to a few types of plastic materials, made on a limited number of species and rather heterogeneous, making it difficult to draw definitive conclusions on the effects of micro- and nanoplastics on CA activity from the in vivo data. However, they allow potential mechanisms to be glimpsed through which micro- and nanoplastics can influence the activity and expression of carbonic anhydrase in vivo in different organisms. These mechanisms involve direct and indirect interactions that affect the enzyme’s structure, function, and the regulatory pathways controlling its expression. Adsorption of the enzyme on the MP/NP surface appears to be a relevant mechanism of the direct interaction of MPs and NPs on the enzyme followed by conformational changes and, in turn, inhibition of the catalytic activity, as indicated by in vitro and in silico studies [82,83,99,100]. Moreover, other mechanisms can be involved in the alteration of CA expression under in vivo exposure which can interfere with the intracellular pathways controlling CA expression. MPs and NPs are known to induce oxidative stress through various mechanisms, including direct ROS generation and the alteration of antioxidant defenses, and triggering inflammatory responses [103,104,105]. This phenomenon has been observed in various studies across different organisms and experimental setups. In turn, oxidative stress has been demonstrated to significantly impact the activity and expression of CA in several organisms and an experimental model [106,107,108]. Therefore, it can be hypothesized that the induction of oxidative stress could represent one of the possible indirect mechanisms by which MPs or NPs can interfere with CA expression in different organisms. Further studies are required to clarify this intriguing aspect of the research.

## 4. CA Esterase Activity: Potentiality for Use in Plastic Degradation

Although CA is an enzyme primarily known for its role in catalyzing the reversible hydration of carbon dioxide, it also exhibits esterase activity in several organisms across different domains of life, including animals, plants, bacteria, and fungi [109,110]. Since the discovery of the esterase activity of CA in the early 1960s [111], several structural, functional, and mutational studies have shown that the hydratase activity and the esterase activity of CA share a similar mechanism, and the same catalytic pocket, implicating that esterase activity is indicative of hydratase activity [112,113]. The esterase activity, while not the primary role of CA, highlights the enzyme’s versatile catalytic capabilities in different biological contexts.

In general, the potential of esterase enzymes to degrade plastics is an area of interest due to the increasing environmental concerns associated with plastic waste. Esterases have shown potential in breaking down various types of plastic polymers, making them a promising tool for bioremediation and recycling efforts. Esterases from several organisms, including bacteria and fungi, have shown the ability to degrade biodegradable plastics by breaking down ester bonds into monomers and oligomers [29,30]. For example, esterases from *Pseudozyma antarctica* and *Paraphoma* sp. *B47–9* can degrade biodegradable plastics like poly(butylene succinate) and poly(butylene adipate) into monomers and oligomers [30], demonstrating the practical application of these enzymes in breaking down plastic materials into smaller, less harmful components. Specific esterases from *Fusarium culmorum* have been identified to degrade the plasticizer di(2-ethylhexyl) phthalate (DEHP) into simpler compounds, suggesting a potential for broader plastic degradation applications [114]. Esterases from *Roseateles depolymerans* strain TB-87 can degrade aliphatic–aromatic copolyesters, indicating their potential to break down complex plastic structures [115]. Moreover, structural studies of microbial esterases revealed that they can hydrolyze ester bonds in polyesters, contributing to the degradation of both aliphatic and semi-aromatic polyesters [116,117].

The state-of-the-art literature indicates that esterase enzymes have significant potential in degrading various types of plastics, including biodegradable polyesters and synthetic polymers like polyurethane and PET. The role of CA in plastic degradation, particularly through its esterase activity, is not well documented in the literature to date. However, the presence of esterase activity in various CA isoforms in nature suggests the potentiality of these enzymes to hydrolyze ester bonds in plastics and pave the way for future studies and potential applications of this widespread enzyme in the field of sustainable plastic degradation technologies.

## 5. Limitations and Challenges

The analysis of the literature on the interaction of MPs/NPs with CA highlights several limitations and challenges in this field, which can stimulate future research.

First, a limited understanding of the mechanisms of interaction of MPs/NPs with CA is available from the literature to date. While in vitro and in silico studies have provided insights into how MPs and NPs interact with CA, the exact molecular mechanisms remain unclear. These mechanisms need further exploration across different CA isoforms and plastic types. Moreover, the available in vivo studies are limited in number, covering only a few species and types of plastic materials. This makes it challenging to draw definitive conclusions about the broader ecological and biological impacts of MPs and NPs on CA activity. The existing studies are heterogeneous, with varying methodologies, exposure conditions, and measured outcomes. This lack of standardization complicates the interpretation of results and the comparison across studies. Most studies focus on polystyrene and polyethylene, which are common but not the only types of plastics present in the environment. Other plastic materials, including those with different chemical properties and surface functionalizations, may interact with CA differently, but these interactions remain underexplored. Many studies focus on short-term exposures, which may not capture the full extent of CA’s response to MPs and NPs. Long-term studies are necessary to understand the chronic effects and potential for bioaccumulation. The potential for MPs and NPs to act as vectors for other pollutants is acknowledged, but studies exploring these synergistic effects are scarce. This represents a significant gap in understanding the cumulative impact on CA activity and expression.

CA shows a wide variety of isoforms across different organisms, which are each potentially interacting differently with MPs and NPs. Understanding this variability is crucial for predicting the environmental and biological impacts of plastic pollution. Different species may exhibit varied responses to plastic exposure, which are influenced by factors such as life cycle, habitat, and physiological processes. The challenge lies in extrapolating findings from model organisms to broader ecological contexts. Some in vitro studies indicate the sensitivity of human CA to MPs and NPs, but the implications for human health are not yet fully explored. This represents a significant challenge, particularly in assessing the long-term risks of plastic exposure to human populations.

Moreover, the esterase activity of CA suggests potential for plastic degradation, but this application is not well documented in the literature. The CA role in hydrolyzing ester bonds in plastics remains largely theoretical with few practical studies demonstrating this capability.

Overall, the analysis of the literature on the interaction of MPs/NPs with CA highlights significant research gaps and challenges that help to identify areas where the current understanding of this topic is incomplete or uncertain; it can inspire more comprehensive and standardized studies in this intriguing research field, guiding researchers toward unexplored or underexplored topics, providing a roadmap for future studies.

## 6. Updates and Perspectives

Limitations and challenges can be useful for drawing updates and stimulating perspective selection on this intriguing research topic. Overall, the sensitivity of CA activity and expression to MPs and NPs exposure seems to be a multifaceted phenomenon influenced by several factors, including the nature of the MPs/NPs, their size, the surface functionalization of the particles, and the time and concentration of exposure. Moreover, the responses appear species-specific and, within the same species, they are related to the specific CA isoforms.

Regarding the chemical nature of the particles, the studies on CA mainly focus on PE and PS. Both are hydrophobic, but polystyrene has an added capability for π–π interactions due to its aromatic phenyl groups. The hydrophobic and π–π interactions make polystyrene conducive to adsorbing proteins with hydrophobic regions or aromatic amino acids. Indeed, polystyrene generally shows stronger protein binding compared to polyethylene whose protein interactions are weaker and purely hydrophobic [118,119,120]. It is known that many CA isoforms contain aromatic amino acids such as phenylalanine, tyrosine, and tryptophan in their amino acid sequences (UniProtKB—P00918 (CAH2_HUMAN). These aromatic amino acids in the protein’s amino acid sequence can influence interactions with other molecules and substrates [121], and in the case of PS MPs/NPs, they can be very important in adsorption processes, as also highlighted by Mojiri et al.’s in silico study [100]. Therefore, the different natures of PS and PE could contribute to the variability observed in the responses of CAs to PS and PE MPs and NPs. In addition, for the same type of plastics, the functionalization of the particle surface can influence the effects of MPs/NPs on carbonic anhydrase (CA). In the studies available on CA, only three studies used functionalized PS NPs by carboxylation or amination. In general, the carboxylation and amination of nanoparticles can significantly influence their ability to interact with biological macromolecules. Indeed, these functionalizations significantly modify the surface charge, hydrophilicity, and the potential for specific electrostatic and hydrogen bonding interactions, altering the surface chemistry of the nanoparticles, which can affect their binding affinity, specificity, and overall interaction dynamics [122]. In the few studies available, a comparison between the functionalized NPs with the corresponding not functionalized NPs is lacking, preventing the quantification of the contribution of NP functionalization to the effects on CA. This aspect of research related to the surface paves the way for future research in this field.

Moreover, MP/NP particle sizes are reported to play an important role in the alteration of several enzymatic activities and gene expression in exposed organisms [52,123,124,125,126]. In general, smaller plastic particles in the nanometric dimension can be more easily absorbed and distributed to all the tissues of the organisms through the circulatory system, reaching specific tissues, while larger plastic particles are in most cases excluded from cellular internalization and can be only trapped in the gills and the digestive tract, as demonstrated in fish [127]. It is therefore plausible to think that NPs might have a greater impact on the activity and expression of CA in vivo compared to MPs. Currently, available studies do not allow for a conclusive statement on this aspect; however, they can represent the base for future research aimed at clarifying the impact of the size of MPs and NPs on the activity and expression of CA in different organisms.

Another factor that can influence the effect of MPs and NPs on CA is the exposure time. Most of the in vivo studies currently available in the literature have been conducted over short exposure times, within 24–96 h, while many more long-term studies are needed to better understand the effects on the activity and expression of this enzyme under exposure conditions closer to real-world scenarios. Additionally, another aspect deserves particular attention. Most studies conducted so far have used commercial NPs, which are generally spherical. In reality, many of the NPs present in the environment come from the degradation of plastic materials and may have different shapes and surfaces that are not necessarily spherical and regular, which are characteristics that can influence the interaction with biological systems. Therefore, in perspective, the future use of MPs/NPs that exhibit characteristics more similar to those of real NPs resulting from environmental degradation processes could produce more environmentally realistic knowledge.

In these last few decades, the study of molecular and cellular effects of pollutants has made important advancements in the development of biologically-based methodologies as environmental “diagnostic” tools for early warning detection of the impact of pollution, which are useful for environmental biomonitoring and risk assessment [128,129,130]. In this field, molecular and biochemical alterations such as changes in enzyme activity and expression in bioindicator organisms are attractive biomarkers of environmental health because they offer a rapid and sensitive tool for monitoring the impact of pollutants on living organisms [52,101,130].

Considering the key physiological roles played by CA in living organisms, it is reasonable to hypothesize that any alteration of CA activity by MPs/NPs exposure could represent a threat to the health status of the organism. Although further research is required in this field, the experimental evidence from in vitro and in vivo studies suggests the possible relevance of CA alterations as biomarkers of exposure/effect. There is still a lot of research needed in this field, but, in perspective, the studies of the toxicological and ecotoxicological effects of MPs and NPs on CAs could be fruitful for the development of CA-based biomarkers of exposure and effect on bioindicator organisms for environmental monitoring application.

Moreover, CA and its esterase activity can open new perspectives for the use of CA or CA-engineered variants with enhanced esterase activity for the degradation process of plastic materials. For example, its potential use in combination with other enzymes that target different types of bonds in plastic polymers could lead to a more comprehensive breakdown of complex plastics, making the recycling process more efficient.

## 7. Conclusions

The investigation into how MPs/NPs affect enzymes such as CA is a relatively new and growing field driven by the increasing recognition of the pervasive presence of MPs and NPs in the environment and their potential to impact biological systems. Studying the impact of MPs/NPs on CA is crucial, because this enzyme plays a vital role in various physiological processes in animals, plants, and bacteria. In vitro studies indicate that MPs and NPs can interact with CA, inhibiting its activity through adsorption and subsequent conformational changes. In vivo studies on various species (mollusks, fish, crustaceans, algae, humans) reveal that MPs/NPs can alter CA gene expression and activity, though results vary based on particle type, size, and exposure conditions. Long-term exposure to MPs/NPs can lead to reduced CA tissue content and gene expression changes, suggesting potential disruptions in physiological processes. The analysis of the currently available literature allows us to draw some knowledge gaps and challenges that can help to identify areas where the current understanding of this topic is incomplete or uncertain and can help to address future research in the field. More long-term in vivo studies are needed to better understand the real-world impact of MPs/NPs on CA activity and expression. Research should focus on environmentally relevant particles with varied shapes and surfaces rather than just commercial spherical NPs. Future studies should also explore the synergistic effects of MPs/NPs with other environmental pollutants to comprehensively assess their impact on CA and related biological processes.

Moreover, the analysis of the available knowledge on the interaction of MPs/NPs with CA allows us to identify future research development perspectives and applications such as the potential use of alterations of CA activity and expression in bioindicator organisms for the development of CA-based biomarkers of exposure and effects on bioindicator organisms for environmental monitoring application. Another intriguing perspective of the interaction between CA and MPs/NPs is the potential use of CA or CA-engineered variants with enhanced esterase activity for the degradation process of plastic materials.

Overall, the review underscores the complex and multifaceted nature of CA’s interactions with MPs and NPs, highlighting the need for further research to elucidate these mechanisms and their broader ecological implications.

## Figures and Tables

**Table 1:** In vitro and in silico studies on the effects of MPs and NPs on carbonic anhydrase.

CA Isoforms	MP or NP Type	IC_50_	MP or NP Size	In Vitro/In Silico	Toxicological Effects on CA	Mechanism of Action	Ref.
HCAII	PS NPs: (PSCOOH, PSNH_2_ PS)	0.3–0.6 nM	PSCOOH (43 nm) PS (46 nm) PSNH2 (59 nm)	in vitro	inhibition	adsorption	[82]
HCAI, HCAII, trHCAII	PSCOOH	-	25, 41, 92, 114 nm	in vitro	inhibition	adsorption	[83]
HCAI, trCAII	PSCOOH	-	52 nm 52 nm	in vitro	-	adsorption	[84]
CA estrinsic protein in *Chaetoceros neogracile*	PS MPs	-	0.1 μm	in silico	-	potential interaction between the aromatic ring of polystyrene and some CA amino acids	[1]

**Table 2:** In vivo studies on the effects of MPs and NPs on carbonic anhydrase.

Species	MP or NP Type	MP/NP Concentrations	MP or NP Size	Other Contaminant Present	Exposure Time	Tissues	Effect on CA	Ref.
*Mytilus coruscus* (bivalve mollusc)	PS MPs	2, 200 μg/L	90 μm	-	-	whole organism	CA gene expression upregulation (transcriptomic analysis)	[85]
*Mytilus coruscus* (bivalve mollusc)	PS MPs	0.26 mg/L	5 μm	carbamazepine (CBZ) 10 μg/L	4 weeks	mantle	CA content reduction (ELISA)	[86]
*Mytilus galloprovincialis* (bivalve mollusc)	PS MPs	50–500 particles mL^−1^	3 μm	-	48 hpf	whole embrio	significantly up-regulation of CA transcripts	[87]
*Macrobrachium nipponense* (river prawn)	PS NPs	0, 5, 10, 20, 40 mg/L	75 nm	-	7, 14, 21, 28 days	gills	CA gene upregulation at 5, 10 and 20 mg/L; no effects at 40 mg/L	[88]
*Alexandrium tamarense* (dinoflagellate)	PS MPs/NPs	5, 50 mg/L	Two sized: 0.1, 1 μm	-	0, 6, 24, 48, 72, 96 h	microalgal suspension	CA_ext_ activity inhibition	[89]
*Laternula elliptica* (bivalve mollusc)	PSCOOH NPs	5 µg/L	62 nm	TiO_2_ (25 nm) 5 µg/L	96 h	gills	CA9 gene expression upregulation when exposed to both PSCOOH NPs and TiO_2_	[90]
*Homo sapiens*	PSCOOH NPs	10^9^ particles mL^−1^	40, 200 nm	-	24 h	embryo Induced pluripotent stem cells (in vitro)	CA4 gene expression upregulation	[91]
*Mytilus galloprovincialis* (bivalve mollusc)	PS-NH_2_	0.150 mg/L	50 nm	-	24, 48	embryos	CA gene expression downregulation after 48 h	[92]
*Pomacea canaliculate* (gastropod mollusc)	PE MPs	20 μg/L	10–90 μm	-	24, 72, 120 h	digestive gland, mantle	CA activity inhibition in digestive gland after 24 h; CA activity stimulation in mantle after 24 and 72 h	[93]
*Prochilodus lineatus* (fish)	PE MPs	20 μg/L	10–90 μm	Copper (Cu) 10 μg/L	24 and 96 h	gills	no significative effect on CA activity	[94]
*Astyanax lacustris* (fish)	LDPE MPs	10 mg/L	100, 200 µm	PAH 2.28 μg/L	96 h	gills	no significative effect on CA activity	[95]
*Microcystis aeruginosa* (cyanobacteria)	PLA MPs	10, 50, 200 mg/L	2.564 μm	-	day 27, 39, 51, 63	cells in suspension	CA content reduction (ELISA)	[96]
